# *S. boulardii* Early Intervention Maintains Gut Microbiome Structure and Promotes Gut Mucosal Barrier Function in Early-Weaned Rats

**DOI:** 10.3390/nu14173485

**Published:** 2022-08-24

**Authors:** Zhipeng Yang, Yanting Wu, Xiangchen Liu, Mei Zhang, Jian Peng, Hongkui Wei

**Affiliations:** 1 Department of Animal Nutrition and Feed Science, College of Animal Science and Technology, Huazhong Agricultural University, Wuhan 430070, China; 2 The Cooperative Innovation Center for Sustainable Pig Production, Wuhan 430070, China

**Keywords:** early-weaned rats, *Saccharomyces boulardii*, gut mucosal barrier, gut microbiome homeostasis, inflammatory responses, microbial metabolism

## Abstract

Early weaning leads to the disorder of the gut microbiome and gut mucosal barrier injury. Early intervention of gut microbiome colonization contributes to the development of the gut microbiome and gut function. The aim of this study was to explore the effects of *Saccharomyces boulardii* (*S. boulardii*) early intervention on the gut microbiome structure and gut mucosal barrier function of early-weaned rats. The results showed that *S. boulardii* early intervention improved growth performance along with a decrease in pathogenic bacteria, an increase in beneficial bacteria, a stable and complex microbiome, and a high level of microbial metabolism. Moreover, *S. boulardii* upregulated the mucosal barrier function including goblet cells and relative gene expression, tight junction, and sIgA level. Furthermore, *S. boulardii* suppressed the inflammatory response and promoted the anti-inflammatory response. Our study may provide a possible early intervention strategy for preventing an early weaning-induced disorder of the gut microbiome and loss of gut mucosal barrier function.

## 1. Introduction

The intestine is not only the digestive organ but also the body’s largest organ of immunity, which is a vital barrier that protects the body from harmful macromolecular invaders such as pathogenic bacteria [1]. Weaning, in particular early weaning, can negatively impact growth performance by breaking the maturation of gut mucosal barriers as a stressful event for mammals [2]. Early weaning can easily result in an immune stress response in the gut, which in turn can lead to bowel diseases, such as diarrhea, infection, dyspepsia, and malnutrition [3]. Moreover, early weaning can lead to the disorder of gut microbial composition, such as the increased abundance of pathogens, *Escherichia Shigella* and *Helicobacter* [4], and the decreased proportion of beneficial bacteria such as *Bifidobacterium*, *Bacteroides*, *Bacillus*, *Lactobacillus*, and *Ruminococcus* [5].

Much research has demonstrated that the gut microbiome serves a crucial role in newborns’ gut mucosal barrier development. The gut microbiome is easily influenced by dietary changes and the environment in early life [6]; thus, neonatal gut microbial initial colonization is an important window of opportunity. Early intervention of gut microbiome colonization contributes to the development of the gut microbiome of neonates and has an impact on host intestine mucosal homeostasis [7,8]. Probiotics are an effective way to modulate gut microecology and maintain intestine mucosal homeostasis which has attracted a lot of interest from researchers [9].

*S. boulardii* is a type of saccharomyces cerevisiae, which belongs to facultative anaerobic fungi and exerts anti-cancer, immune modulation, antibacterial, antiviral, and antioxidant functions in the body [10]. Supplementation with *S. boulardii* in early life reduced diarrhea duration and severity and promoted growth performance and shaped the fecal microbial in the neonatal piglets [11,12]. Compared to the impact of early-life intervention using *S. boulardii* on the early weaning rat models including the gut microbial structure and metabolites, gut mucosal barrier functions such as goblet cells and the tight junction are not clear. Here, we constructed a model of early-weaned rats to investigate the impact of *S. boulardii* early intervention on the gut mucosal barrier functions’ injury and disorder of the gut microbiome from early weaning.

## 2. Materials and Methods

### 2.1. Animals

Wistar rats obtained from the Animal Experiment Center at Hubei Disease Prevention and Control Center (Wuhan, China) were used for the present study. Animal welfare and experimental procedures were monitored according to the Guide FOR THE CARE AND USE OF LABORATORY ANIMALS (Eighth Edition) and were approved by the ethical committee in Huazhong Agricultural University (Approval Code: HZAURA-2022-0016). The rats were housed under specific pathogen-free conditions in a temperature-controlled room at 23 ± 2 °C. There was free access to food and water.

### 2.2. Experimental Design

**Figure 1:**
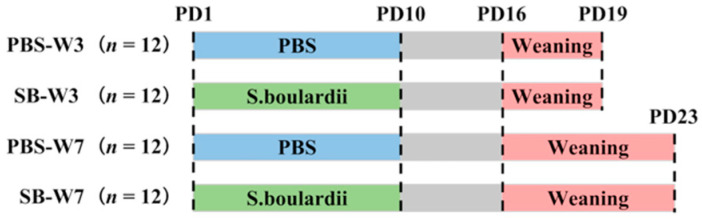
Experimental design. We chose 6 pregnant Wistar rats and monitored them daily until parturition. After birth, each litter was culled to a size of 8 pups. On PD1, pups were randomly divided into two groups (PBS or SB) in each litter. Rats in the PBS groups were fed with PBS once per day from PD1 to PD10; rats in the SB groups were fed with 1 × 10^9^ CFUs of *S. boulardii* once per day from PD1 to PD10. All pups were weaned on PD16. On PD19, 4 rats from every litter from two groups were randomly chosen and were asphyxiated by CO_2_ inhalation and sacrificed. On PD23, the rest of the rats were asphyxiated by CO2 inhalation and sacrificed.

We chose 6 pregnant Wistar rats and monitored them daily until parturition. After birth, each litter was culled to a size of 8 pups, with 4 pups of each sex. On postnatal day 1 (PD1), pups were randomly divided into two groups (PBS or *S. boulardii*) in each litter. Rats in the PBS groups were fed with PBS once per day from PD1 to PD10; rats in the SB groups were fed with 1 × 10^9^ colony-forming units (CFUs) of *S. boulardii* once per day from PD1 to PD10. The test product was Levucell SB^®^ (Lallemand SAS, Blagnac, France). All pups were weaned on PD16. On PD19 (day 3 after weaning), 4 rats from every litter from two groups were randomly chosen, with 2 rats of each sex, and were asphyxiated by CO_2_ inhalation and sacrificed. On PD23 (day 7 after weaning), the rest of the rats were asphyxiated by CO_2_ inhalation and sacrificed (Figure 1).

### 2.3. Growth Performance

During the whole trial, each rat’s body weight (BW) was measured, and the feed intake was recorded after weaning to calculate the average daily gain (ADG), average daily feed intake (ADFI), and feed conversion ratio (FCR).

### 2.4. Sample Collection

On day 3 and day 7 after weaning, colonic tissues were collected from all rats and fixed in 4% paraformaldehyde fix solution, and colonic tissues were collected and stored at −80 °C for RNA and protein isolation. Colonic contents were immediately transferred to microcentrifuge tubes and stored at −80 °C until further processing.

### 2.5. Fecal Microbiota Sequence

The colonic contents of rats on day 3 and day 7 after weaning were detected for microbial composition. The method was previously described by Xia et al. [13]. Briefly, a QIAamp DNA Stool Mini Kit (Qiagen Ltd., Frankfurt, Germany) was used to extract the total microbial genomic DNA from colonic contents following the manufacturer’s instructions. Amplicon libraries were sequenced on an Illumina MiSeq (Illumina, Santiago, MN, USA) at the Personalbio, Shanghai, China. QIIME 2 2019.4 was used to perform the microbiome bioinformatics [14]. DADA2 plugin was used to filter quality, denoise, merge and remove chimera for sequences [15]. To assign taxonomy to ASVs, the classify-sklearn naïve Bayes taxonomy classifier in the feature-classifier plugin was used [16] against the Greengenes 13_8 99% operational taxonomic unit reference sequences [17]. The Chao1, Shannon, Simpson, and Observed species values were used to assess the diversity of alpha. Beta diversity measures depended on Bray–Curtis distance.

### 2.6. Co-Occurrence Network Diagram

The differences in the abundance were analyzed by Spearman’s rank correlation analysis. The genus-level species were selected with presence in >3 of the 7 samples in each group. Co-occurrence networks were constructed using data with correlation coefficients |r-value| > 0.5 and *p*-values < 0.05. Cytoscape (v3.9.1) was used for visualization and network analysis. The modules were divided by the MCODE in Cytoscape.

### 2.7. Short-Chain Fatty Acids Determination

The SCFA concentrations of colonic contents were analyzed through a gas chromatographic method previously described by Wang et al. (2022) with slight modifications [18]. Briefly, 0.5 g of colonic contents was homogenized in 1 mL of deionized water. The samples were centrifuged at 12,000× *g* at 4 °C for 10 min. We then acidified the supernatants (0.1 mL) with 25% metaphosphoric acid at a 1:5 ratio for 30 min on ice. Then, the sample was centrifuged at 12,000× *g* for 10 min to obtain the supernatant. The supernatant was extracted with an equal volume of ethyl acetate. After that, it was centrifuged at 12,000× *g* at 4 °C for 10 min, and the supernatant was collected. The sample was injected into a GC 3000 series gas chromatograph (Thermo, Waltham, MA, USA) equipped with an Omega wax 250 column 30.0 m × 0.25 mm × 0.25 μm (Sigma, St. Louis, MO, USA).

### 2.8. Hematoxylin and Eosin (HE) Staining

Firstly, we deparaffinized and hydrated them to water then immersed the slides in hematoxylin solution for 3 to 5 min and rinsed them in water. Then, differentiate sections with acid alcohol (Sinopharm, Beijing, China), rinse again, and blue up sections with ammonia solution and washed in slowly running tap water. Finally, stain in eosin and dehydrate and mount. The section was scanned with Pannoramic SCAN (3DHISTECH CaseViewer, Budapest, Hungary). The morphology and histological score were analyzed by CaseViewer software (3DHISTECH CaseViewer, Budapest, Hungary). We assessed the distance from the apical side to the basal side of the crypt base on at least 10 intact and well-oriented crypts of each sample.

### 2.9. Periodic Acid Schiff (PAS) Staining

We deparaffinized and hydrated to water. We stained in periodic acid: we immersed slides in periodic acid for 15 min and washed in tap water 2 times. Then, we washed the slides in distilled water 2 times. We stained in Schiff’s reagent: we immersed the slides in Schiff’s reagent for 30 min (in the darkroom) and washed in slowly running tap water for 5 min. The section was scanned with Pannoramic SCAN (3DHISTECH CaseViewer, Budapest, Hungary). We assessed the number of goblet cells in three different parts of 1 square millimeter per section.

### 2.10. RNA Isolation and Quantitative Real-Time PCR

Total RNA was extracted using the TRIzol reagent (Keepbio, Taiwan, China), then we detected the concentration of RNA by NanoDropfi ND-1000 Spectrophotometer (Thermo, Waltham, MA, USA) and transcribed it into cDNA using reverse transcriptase (Aidlab, Beijing, China). Quantitation of the mRNA levels by quantitative real-time (qPCR) was performed on a real-time PCR system (Bio-Rad, Hercules, CA, USA) using SYBR Green qPCR Master Mix (Bimake, Houston, TX, USA). The sequences of the primers were listed in Table 1.

### 2.11. Western Blot Analysis

The colonic tissues were extracted with RIPA Lysis Buffer (Beyotime, Shanghai, China) supplemented with 100X Protease Inhibitor Cocktail (BBI, Shanghai, China). The protein concentration was determined using the BCA Kit (Beyotime, Shanghai, China). We took 40 μg of the protein sample and added 4 × sample buffer (Sanggon Biotech, Shanghai, China). Proteins were separated on a 12% polyacrylamide precast SDS gel (ACE Biotechnology, Xiangtan, China) followed by blotting on PVDF membranes (MERCK, Kenilworth, NJ, USA). The membrane was blocked with TBST buffer (Monad, Wuhan, China) containing 5% skim milk powder (BBI, Shanghai, China) for 1 h and then incubated with the primary antibody overnight at 4 °C: anti-β-actin (1:50,000, Abclonal, Wuhan, China), anti-Claudin-1 (1:1000, Affinity, Shanghai, China), and anti-Occludin (1:1000, Affinity, Shanghai, China). Secondary antibodies, anti-mouse, or rabbit IgG-HRP (1:30,000, Abclonal, Wuhan, China) were used to detect primary antibodies. Binding was detected using an enhanced chemiluminescence detection kit (Affinity, Shanghai, China) according to the manufacturer’s instructions. Densitometry was performed using Image-J software (Bethesda, Rockville, MD, USA).

### 2.12. ELISA

The colonic tissues (50 mg) and 200 uL of PBS were added to the EP tube, and then they were pulverized with a homogenizer. Then, they were centrifuged at 12,000 rpm for 10 min at 4 °C, and the supernatant was collected for the Elisa assay. Before the Elisa assay, the protein concentration was determined using the BCA Kit (Beyotime, Shanghai, China). Rat ELISA kits including interleukin 1β (IL-1β) (2R-KMLJr30206), interleukin 6 (IL-6) (2R-KMLJr30219), tumor necrosis factor-α (TNF-α) (2R-KMLJr31063), and secretory immunoglobulin A (sIgA) (2R-KMLJr30356) were purchased from Nanjing Camilo biological engineering Co., Ltd. (Camilo, Nanjing, China) to determine the concentrations of cytokines in the colon and serum according to the manufacturer’s instructions.

### 2.13. Bacterial Load Assay

To quantitatively determine the bacterial load in different organs, approximately 100 mg of the sample was resuspended in a sterile EP tube with 1 mL of PBS and recorded 10^−1^. We added 20 μL of the suspension to 200 μL of PBS and recorded 10^−2^. This was followed by plating onto LB medium using serial dilutions. After incubation for 12–16 h, the counting of CFUs occurred.

### 2.14. Statistical Analysis

Data were analyzed using GraphPad Prism version 9.0.0 software (San Diego, CA, USA) and expressed as the means ± standard error of the mean (SEM). Firstly, data were assessed for the normality of the distribution by the Shapiro−Wilk test. Then, a t-test was used to analyze the data with a normal distribution, and the data with a non-normal distribution were analyzed by the Mann−Whitney U test. The Wilcoxon rank-sum test was used for the analysis of microbial differences. Statistical significance was defined as *p* < 0.05. Graphical representations were used by GraphPad Prism version 9.0.0 software (San Diego, CA, USA) and Majorbio Cloud Platform (Majorbio, Shanghai, China). The graphical abstract was conducted by Figdraw.

## 3. Results

### 3.1. Effects of S. boulardii Early Intervention on the Growth Performance of Early-Weaned Rats

To evaluate the effects of *S. boulardii* early intervention on the growth performance, BW, ADG, and ADFI of early-weaned rats were measured (Table 2). Suckling rats given *S. boulardii* in early life did not change BW before weaning compared to the PBS group (*p* = 0.84). Moreover, *S. boulardii* early intervention had a trend to increase the BW on day 7 (*p* = 0.07). Further, *S. boulardii* early intervention significantly increased ADG during day 3~7 (*p* = 0.02) after weaning. There was no difference in ADFI between the PBS and SB groups after weaning (*p* > 0.05). More importantly, *S. boulardii* early intervention tended to raise FCR during day 3~7 (*p* = 0.09) after weaning.

### 3.2. Effects of S. boulardii Early Intervention on the Gut Microbial Diversity

**Figure 2:**
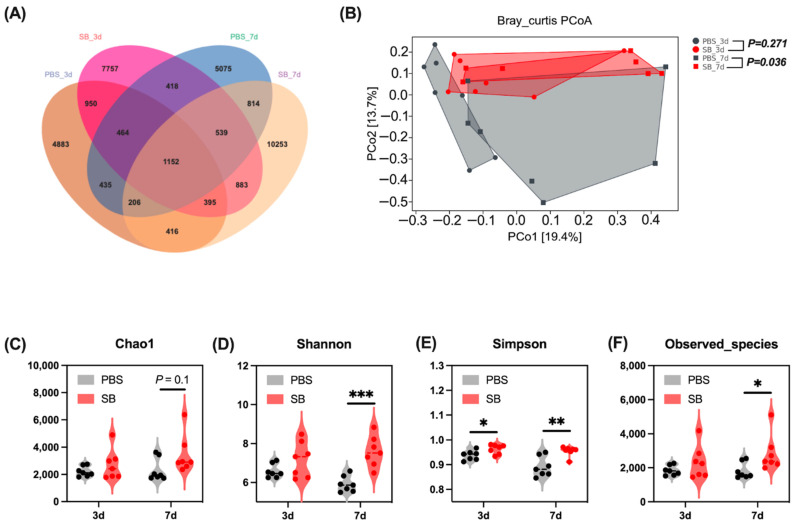
Effects of *S. boulardii* early intervention on the gut microbial diversity. (**A**) Venn diagram of core operational taxonomic units in the colonic content. (**B**) β-diversity of PCoA based on Bray–Curtis distances. α-diversity including Chao1 index (**C**), Shannon index (**D**), Simpson index (**E**), and Observed_species (**F**). Values are expressed as means ± SEM, *n* = 7, * *p* < 0.05, ** *p* < 0.01, *** *p* < 0.001. d, day. PBS, rats early intervened with phosphate buffer solution. SB, rats early intervened with S. *boulardii*. *S. boulardii* early intervention significantly changed the phenotype of the gut microbiome.

A total of 6035, 8909, 6227, and 11,405 operational taxonomic units (OTUs) were separately obtained from the PBS-3d group, SB-3d group, PBS-7d group, and PBS-7d group, respectively, and 1152 OTUs were common in the four groups (Figure 2A). Moreover, there were 4883, 7757, 5075, and 10,253 OTUs uniquely identified from the PBS-3d group, SB-3d group, PBS-7d, group, and PBS-7d group, respectively (Figure 2A).

The effects of *S. boulardii* early intervention on colonic content microbiota α and β diversity were measured. On day 3 after weaning, *S. boulardii* early intervention significantly increased the Simpson index (*p* < 0.05) (Figure 2E), whereas it had no effects on the Chao1 index, Shannon index, and Observed_species (Figure 2C,D,F). On day 7 after weaning, the α-diversity index including Shannon and Simpson indexes and Observed_species was significantly increased in the SB group (*p* < 0.05) (Figure 2D–F); furthermore, *S. boulardii* early intervention had a trend to increase the Chao1 index (*p* = 0.1) (Figure 2C). For the β-diversity, principal coordinates analysis (PCoA) based on the Bray–Curtis distances showed significant differences between the PBS group and the SB group on day 7 after weaning (*p* < 0.05) but not on day 3 after weaning (Figure 2B).

**Figure 3:**
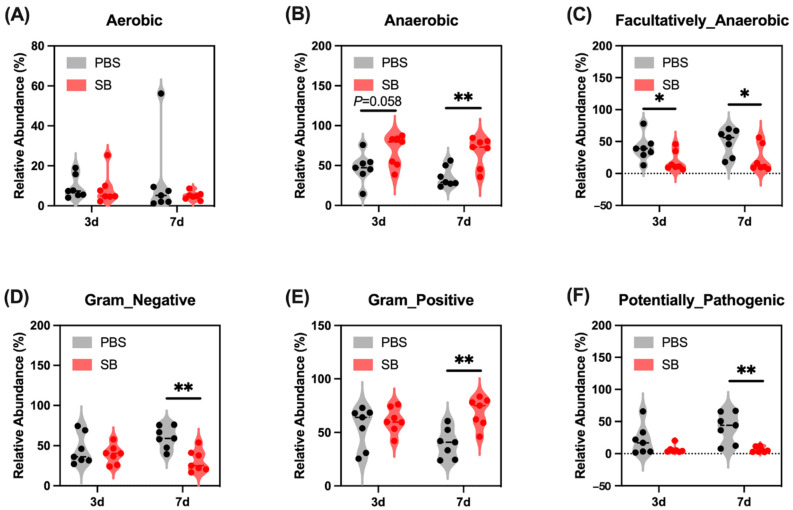
Effects of *S. boulardii* early intervention on the phenotype of the gut microbiome. BugBase analysis for Aerobic (**A**), Anaerobic (**B**), Facultatively_Anaerobic (**C**), Gram_Negative (**D**), Gram_Positive (**E**), and Potentially_Pathogenic (**F**). *n* = 7 * *p* < 0.05, ** *p* < 0.01. d, day. PBS, rats early intervened with phosphate buffer solution. SB, rats early intervened with *S. boulardii*.

Next, we conducted the BugBase analysis to further investigate the feature of the colonic content microbiome. We found that on day 3 after weaning, *S. boulardii* early intervention had a trend to increase the relative abundance of Anaerobic (Figure 3B) and significantly decrease the relative abundance of Facultatively_Anaerobic (*p* < 0.05) (Figure 3C) but had no influence on the relative abundance of Aerobic, Gram_Negative, Gram_Positive, and Potentially_Pathogenic (Figure 3A,D–F, respectively). On day 7 after weaning, the relative abundance of Facultatively_Anaerobic, Gram_Negative, and Potentially_Pathogenic were significantly lower (Figure 3C,D,F, respectively) and the relative abundance of Anaerobic and Gram_Negative were markedly higher (Figure 3B,D, respectively) in the SB group than in the PBS group.

### 3.3. Effects of S. boulardii Early Intervention on the Composition and Structure of the Gut Microbiota

**Figure 4:**
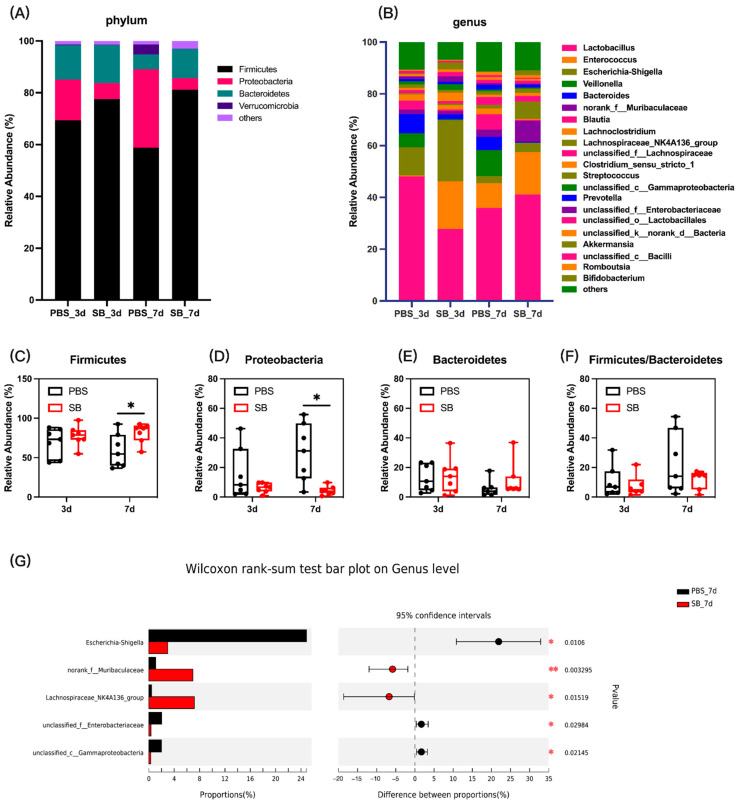
Effects of *S. boulardii* early intervention on the colonic content microbiota composition. (**A**) Microbial composition at the phylum. (**B**) Microbial composition at the genus. The relative abundance of Firmicutes (**C**), Bacteroides (**D**), Proteobacteria (**E**), and the ratio of Firmicutes and Bacteroidetes (**F**). (**G**) Differential microbial composition at the genus level based on the Wilcoxon rank sum test. Values are expressed as means ± SEM, *n* = 7 * *p* < 0.05, ** *p* < 0.01. d, day. PBS, rats early intervened with phosphate buffer solution. SB, rats early intervened with *S. boulardii*.

Next, we measured the colonic content microbiota composition. At the phylum level, the bar plot showed that Firmicutes, Proteobacteria, and Bacteroidetes were the main three dominant phyla in both the PBS group and SB group (Figure 4A). The relative abundance of Firmicutes in the SB group increased (*p* < 0.05) (Figure 4C), and the relative abundance of Proteobacteria decreased (*p* < 0.05) (Figure 4D) on day 7 after weaning compared with the PBS group. However, the relative abundance of Firmicutes and Proteobacteria had no significant change between the PBS and SB groups on day 3 after weaning (*p* > 0.05) (Figure 4C,D). Moreover, there was not a significant difference in the relative abundance of Bacteroidetes and the ratio of Firmicutes and Bacteroidetes between the PBS and SB groups (Figure 4E,F). At the genus level, data showed that *S. boulardii* early intervention significantly decreased the relative abundance of *Shigella* (*p* < 0.05), *unclassified_f__Enterobacteriaceae* (*p* < 0.05), and *unclassified_c__Gammaproteobacteria* (*p* < 0.05) and increased the relative abundance of *norank_f__Muribaculaceae* (*p* < 0.01) and *Lachnospiraceae_NK4A136_group* (*p* < 0.05) (Figure 4G) on day 7 after weaning, whereas there were no significant changes on day 3 after weaning.

### 3.4. Effects of S. boulardii Early Intervention on Microbial Co-Occurrence Patterns

**Figure 5:**
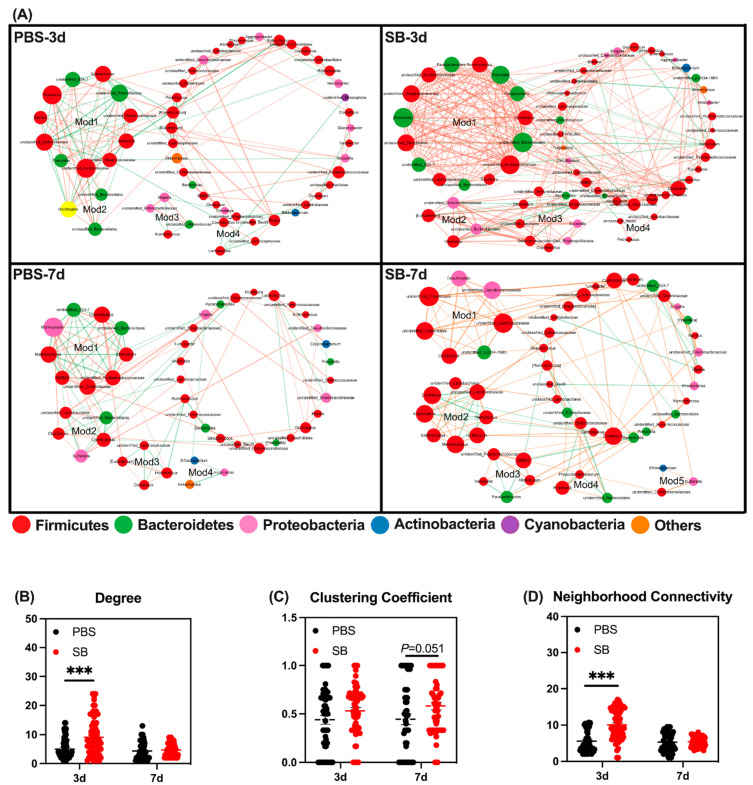
Effects of *S. boulardii* early intervention on microbial co-occurrence patterns. (**A**) Microbial co-occurrence networks of 4 groups. The size of each node is proportional to the degree of correlation. Phylum information for each node is presented in a different color. The edges represent the correlation between the two genera and the thickness of the edge represents the strength of the correlation, where orange represents a positive correlation and green represents a negative correlation. (**B**) Degree of co-occurrence networks for bacterial communities. (**C**) Clustering coefficient of co-occurrence networks for bacterial communities. (**D**) Neighborhood connectivity of co-occurrence networks for bacterial communities. Values are expressed as means ± SEM, *** *p* < 0.001. d, day. PBS, rats early intervened with phosphate buffer solution. SB, rats early intervened with *S. boulardii*.

Next, we conducted co-occurrence networks in the four groups. There were four, four, four, and five modules of co-occurring microbial members in the PBS-3d, SB-3d, PBS-7d, and SB-7d groups, respectively (Figure 5A). These four groups were also compared with respect to their network property parameters and node-level topological features. The network property parameters are shown in Table 3. At the node level, the degree and neighborhood connectivity of the SB group was significantly higher than the PBS group (*p* < 0.001) on day 3, but there was no difference between the two groups on day 7 (Figure 5B,D). Moreover, the clustering coefficient in the SB group tended to rise compared with the PBS group on day 7 (*p* = 0.051) (Figure 5C). These results indicated that *S. boulardii* early intervention strengthened tight connections and interactions between bacteria, the centrality of the network, and the degree of community modularity.

### 3.5. Effects of S. boulardii Early Intervention on Microbial Metabolites

**Figure 6:**
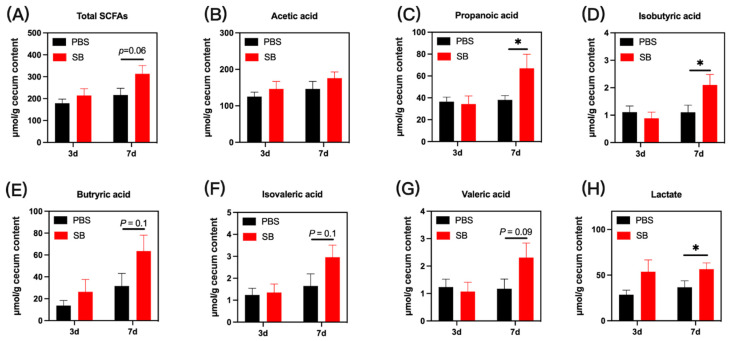
Effects of *S. boulardii* early intervention on microbial metabolites. Microbial metabolites including total SCFAs (**A**), acetic acid (**B**), propanoic acid (**C**), isobutyric acid (**D**), butyric acid (**E**), isovaleric acid (**F**), valeric acid (**G**), and lactate (**H**). Values are expressed as means ± SEM, *n* = 12 * *p* < 0.05. d, day. PBS, rats early intervened with phosphate buffer solution. SB, rats early intervened with *S. boulardii*.

The bacterial metabolites in colon content are presented in Figure 6. On day 3 after weaning, no significant difference was observed in the concentrations of microbial metabolites in the colon content of early-weaned rats in the PBS group and SB group (*p* > 0.05). On day 7 after weaning, the concentrations of propanoic acid, isobutyric acid, and lactate were significantly increased in the colon content in the SB group (*p* < 0.05) (Figure 6C,D,H, respectively). Moreover, rats from the SB group had increased total SCFAs (*p* = 0.06) (Figure 6A), butyric acid (*p* = 0.1) (Figure 6E), isovaleric acid (*p* = 0.1) (Figure 6F), and valeric acid (*p* = 0.07) (Figure 6G). Meanwhile, there was no difference in acetic acid between the PBS group and the SB group.

### 3.6. Effects of S. boulardii Early Intervention on the Gut Morphology Integrity

**Figure 7:**
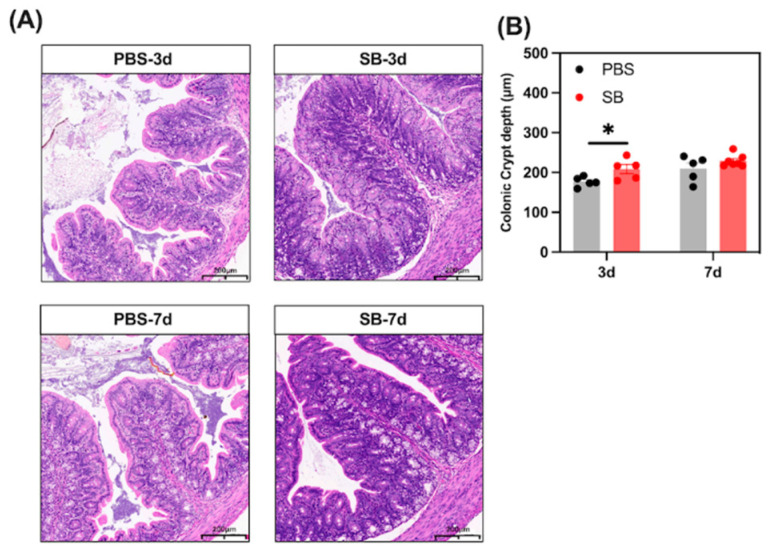
Effects of *S. boulardii* early intervention on the gut morphology integrity. (**A**) Representative images of HE-stained colon tissues (scale bars, 200 μm). (**B**) Statistical analysis of crypt depth. Values are expressed as means ± SEM, *n* = 5–6. * *p* < 0.05. d, day. PBS, rats early intervened with phosphate buffer solution. SB, rats early intervened with *S. boulardii*.

We analyzed the histological morphology of jejunum and colon in the rats (Figure 7). *S. boulardii* early intervention significantly prevented the shortage of crypt depth of the colon on day 3 after weaning (*p* < 0.05). However, there was no change in crypt depth of the colon between the PBS and SB groups (*p* > 0.05). In summary, the findings indicate that *S. boulardii* early intervention promoted gut morphology integrity in early-weaned rats.

### 3.7. Effects of S. boulardii Early Intervention on the Gut Mucosal Barrier Function

**Figure 8:**
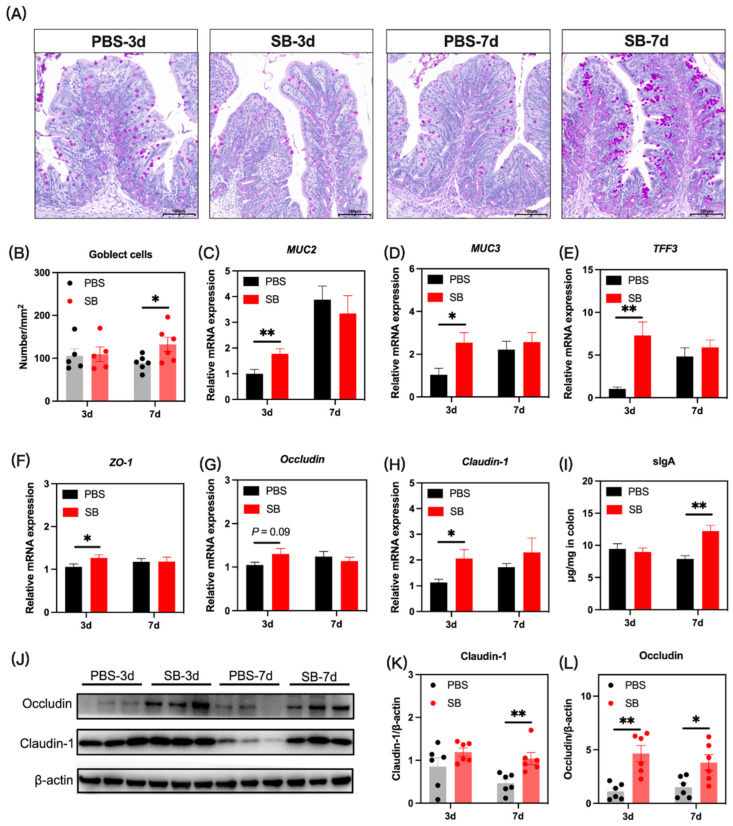
Effects of *S. boulardii* early intervention on the mucosal barriers. (**A**) Representative images of PAS-stained colonic sections. Scale bar = 200 μm. (**B**) The number of goblet cells in the colon. The relative mRNA expression of the gene for MUC2 (**C**), MUC3 (**D**), TFF3 (**E**), ZO-1 (**F**), Occludin (**G**), and Claudin-1 (**H**). (**I**) The concentration of sIgA in the colon. (**J**) The protein expression of Claudin-1 (**K**) and Occludin (**L**). Values are expressed as mean ± SEM, *n* = 5–12. * *p* < 0.05, ** *p* < 0.01. d, day. PBS, rats early intervened with phosphate buffer solution. SB, rats early intervened with *S. boulardii*. MUC2, mucin2. MUC3, mucin2. TFF3, trefoil factor family 3. ZO-1, zonula occluden 1. sIgA, secretory immunoglobulin A.

The gut mucosal barriers in the colon of rats were evaluated to define gut development. We investigated the influence of *S. boulardii* early intervention on the gut mucus by PAS staining, and as shown in Figure 8A,B, the number of goblet cells was significantly increased in the SB group on day 7 after weaning (*p* < 0.05) but not on day 3 after weaning (*p* > 0.05). Next, the relative mRNA expression of mucus-associated genes including *MUC2*, *MUC3*, and *TFF3* was measured. Results showed that the relative mRNA expression of *MUC2*, *MUC3,* and *TFF3* significantly increased in the SB group on day 3 (*p* < 0.05) after weaning but not on day 7 after weaning (*p* > 0.05) (Figure 8C–E, respectively). The tight junction gene was also investigated. The relative mRNA expression of *ZO-1* and *Claudin-1* in the colon was significantly increased in the SB group (*p* < 0.05), and the relative mRNA expression of *Occludin* in the SB group tended to rise (*p* > 0.05) on day 3 after weaning (Figure 8F–H, respectively). There was no significant change in the tight junction gene expression between the SB group and PBS group on day 7 after weaning (Figure 8F–H). At the protein level (Figure 3J), *S. boulardii* early intervention significantly increased the level of Claudin-1 on day 7 after weaning (*p* < 0.01) (Figure 8K) and the level of Occludin on both day 3 and day 7 after weaning (*p* < 0.05) (Figure 8L). Further, the level of sIgA in the colon increased markedly on day 7 after weaning but not on day 3 after weaning (Figure 8I).

### 3.8. Effects of S. boulardii Early Intervention on the Translocation of Bacteria

**Figure 9:**
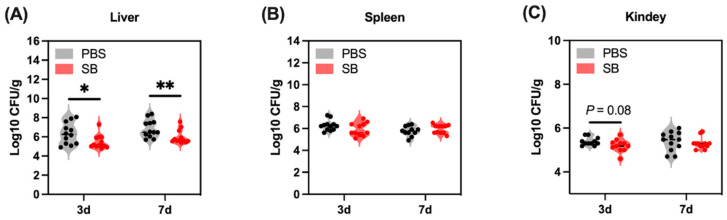
Effects of *S. boulardii* early intervention on the translocation of bacteria. Bacteria load in the liver (**A**), spleen (**B**), and kidney (**C**). Values are expressed as means ± SEM, *n* = 10–12. * *p* < 0.05, ** *p* < 0.01. d, day. PBS, rats early intervened with phosphate buffer solution. SB, rats early intervened with *S. boulardii*.

Gut bacteria translocate to other organs because the injured gut barrier functions after weaning. In the present study, we examined the bacteria load in the liver, spleen, and kidney (Figure 9). The bacteria load in the liver of the SB group was significantly lower than the PBS group on day 3 (*p* < 0.05) and day 7 (*p* < 0.01) after weaning (Figure 9A). Moreover, there was a decreasing trend in the SB group compared with the PBS group on day 3 after weaning (*p* = 0.08) (Figure 9C). Meanwhile, the bacteria load in the spleen between the SB and PBS group did not show a significant change (*p* > 0.05) (Figure 9B).

### 3.9. Effects of S. boulardii Early Intervention on the Inflammatory Response

**Figure 10:**
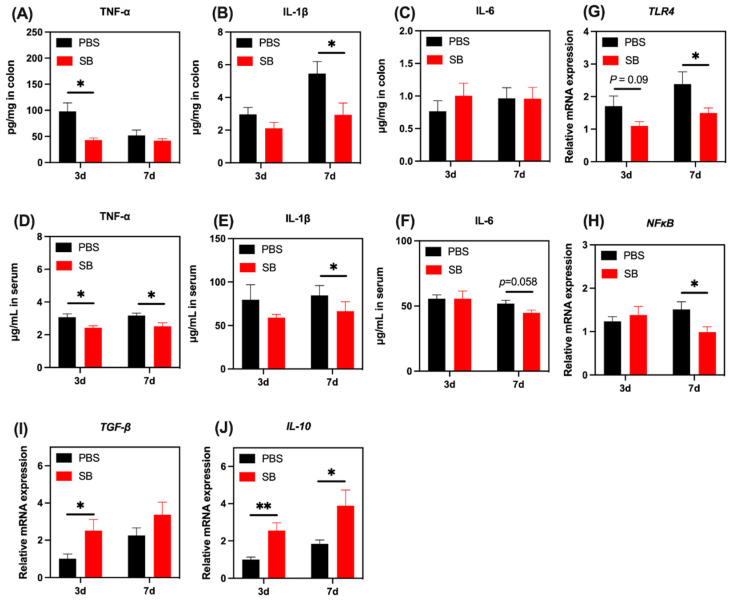
Effects of *S. boulardii* early intervention on the inflammatory response. The levels of TNF-α (**A**), IL-1β (**B**), and IL-6 (**C**) in the colon. The levels of TNF-α (**D**), IL-1β (**E**), and IL-6 (**F**) in serum. The relative mRNA expression of the gene for TLR4 (**G**), NF-κB (**H**), TGF-β (**I**), and IL-10 (**J**). Values are expressed as means ± SEM, *n* = 10–12. * *p* < 0.05, ** *p* < 0.01. d, day. PBS, rats early intervened with phosphate buffer solution. SB, rats early intervened with *S. boulardii*. TNF-α, tumor necrosis factor α. IL-1β, interleukin 1β. IL-6, interleukin 6. TLR4, toll-like receptor 4. NF-κB, nuclear factor kappa-light-chain-enhancer of activated B cells. TGF-β, transforming growth factor β. IL-10, interleukin 10.

We next investigated the levels of several markers associated with the inflammatory response. Pro-inflammatory cytokine TNF-α increased markedly in the colon and serum on day 3 after weaning (*p* < 0.05) but not on day 7 after weaning (Figure 10A,D). Pro-inflammatory cytokines IL-1β in the colon and serum increased markedly on day 7 after weaning but not on day 3 after weaning (Figure 10B,E). Moreover, *S. boulardii* early intervention had a trend to decrease pro-inflammatory cytokine IL-6 in serum on day 7 after weaning (*p* = 0.058) (Figure 10F). The relative mRNA expression of TLR4 and NF-κB were lower in the colon of rats from the SB group than those in the PBS group on day 7 after weaning (Figure 10G,H, respectively). Moreover, there was a decreasing trend in the relative mRNA expression of TLR4 in the SB group compared with the PBS group on day 3 after weaning (*p* = 0.09) (Figure 10G). Next, we investigated the relative mRNA expression of anti-inflammatory cytokines TGF-β and IL-10. As shown in Figure 10I,J, respectively, the relative mRNA expression of TGF-β and IL-10 in the colon of rats from the SB group was significantly higher than that in the PBS group on day 3 after weaning (*p* < 0.05). On day 7 after weaning, the relative mRNA expression of IL-10 in the SB group significantly decreased compared with the PBS group (*p* < 0.05) (Figure 10J).

## 4. Discussion

Weaning, especially early weaning, exerts a negative impact on growth performance, development of gut mucosal barriers, and gut microbiome homeostasis for mammals [19]. Early life is a vital period for microbial colonization and the gut mucosal barrier maturation. Moreover, emerging research reported that microbial colonization contributes to gut mucosa development [19]. Thus, early microbial intervention is a good strategy to shape gut mucosal integrity and functions [20]. *S. boulardii* is a type of saccharomyces cerevisiae that exerts a beneficial effect on the growth performance and gut mucosal barrier and promotes gut microbiota development in sucking piglets [21,22]. Moreover, our previous study confirmed that early-life intervention using *S. boulardii* combined with fecal microbiota transplantation can promote gut microbiota maturation, regulate the immune system, and alleviate weaning stress in piglets [23,24]. Thus, to emphasize the advantages of early microbial intervention for early weaning, we research the effect of *S. boulardii* early intervention after birth on the gut mucosal barrier functions and gut microbiome in early-weaned rats. The results had shown that *S. boulardii* early intervention improved the growth performance of weaned rats during day 3–7 and 1–7 after weaning.

The composition and structure of the gut microbiome is a vital factor for health, which serves an important role in the absorption of nutrients [25], regulating the inflammation response [26], the development of gut mucosal barrier function [27], and so on. Recent research displayed that early weaning leads to a strong disorder of the microbiome in piglets [28]; thus, we explored the effect of *S. boulardii* early intervention on the gut microbiome after early weaning. Microbial diversity is important in all ecosystems to facilitate stability and performance [29], and gut microbial richness and diversity decreased and the microbial community changed after early waning [5]. Our results showed that *S. boulardii* early intervention improved the α-diversity including the Chao1 index, Shannon index, Simpson index, and Observed_species. A previous study also reported that *S. boulardii* early intervention significantly raised α-diversity such as Shannon diversity in the colon content of 2-week weaned piglets [30]. Moreover, the β-diversity showed that *S. boulardii* early intervention deeply shifted the microbial community in rats. Moreover, we predicted phenotypes of the gut microbiome by BugBase analysis, and results displayed that *S. boulardii* early intervention decreased the abundance of Facultatively_Anaerobic, Gram_Negative, and Potentially_Pathogenic and increased the abundance of Anaerobic and Gram_Positive after early weaning.

As for the microbial composition, at the phylum level, we found that there was a higher abundance of Firmicutes and a lower abundance of Proteobacteria in the SB group than in the PBS group, which has a similar trend to the results of the research conducted by Brousseau et al. which showed that *S. boulardii* early intervention increased the relative abundance of Firmicutes and had a trend to decrease the relative abundance of Proteobacteria in the ileum content of 2-week weaned piglets [30]. At the genus level, the decreased genera *Shigella*, *unclassified_f__Enterobacteriaceae*, and *unclassified_c__Gammaproteobacteria* and the increased genera *norank_f__Muribaculaceae* and *Lachnospiraceae_NK4A136_group* were detected after *S. boulardii* early intervention. *Shigella* is a pathogen that easily induces diarrhea [31]. The *Enterobacteriaceae* family includes a large number of pathogenic bacteria, such as *Klebsiella*, *Enterobacter*, *Citrobacter*, *Salmonella*, *Escherichia coli*, *Shigella*, *Proteus*, and *Serratia* [32]. Additionally, the family *Muribaculaceae* was previously known as S24-7, and major mucin monosaccharide foragers impede *Clostridioides difficile*’s access to these mucosal sugars and impair pathogen colonization [33]. It has been demonstrated that *Lachnospiraceae_NK4A136_group* has the potential to be probiotic as a potential butyrate producer [34] and improve gut barrier function in aging rats [35]. A changing microbial composition results in an unstable microbial network, which is usually associated with poor host health [36]. Thus, in this study, we conducted the co-occurrence analysis to investigate the differences in network associations between the SB and PBS groups. Data showed that there were more modules in the SB group than in the PBS group on day 7, and the nodes and edges in the SB group were higher than in the PBS group. Early weaning led to lower degree and clustering coefficient scores [5], and we found that *S. boulardii* early intervention increased the degree, clustering coefficient, and neighborhood connectivity. Therefore, *S. boulardii* early intervention strengthened tight connections and interactions between species, the centrality of the network, and the degree of community modularity. Thus, our results indicated that *S. boulardii* early intervention reduced the pathogens, increased the probiotics, and maintained the homeostasis of the gut microbiome.

It has been shown that metabolites from the gut microbiome can mediate gut microbiome-host interactions. SCFAs are the main metabolites of the gut microbiome and exert anti-inflammatory effects [37] and maintain a gut mucosal barrier function in the gut [38]. Here, we observed that *S. boulardii* early intervention remarkedly increased the content of propanoic acid and isobutyric acid and had a trend to raise total SCFAs, butyric acid, isovaleric acid, and valeric acid. This may be due to the increased abundance of Firmicutes and *Lachnospiraceae_NK4A136_group*, which has been demonstrated to produce SCFAs [34,39].

A severe inflammation response makes the crypt become shorter in the colon [40]. In our study, data showed that *S. boulardii* early intervention significantly prevented the shortage of crypt depth on day 3 after weaning. Gut mucus and tight junctions play a crucial role in maintaining the integrity of the gut mucosal barrier [41]. Early weaning greatly depleted goblet cells and reduced the expression of mucin-related genes and tight junction-related genes in piglets [4]. Thus, we measured the number of goblet cells and the relative mRNA expression of mucin-related genes and tight junction-related genes in the colon, and we found that *S. boulardii* early intervention significantly increased the number of goblet cells and the relative mRNA expression of *MUC2*, *MUC3*, *TFF3*, *ZO-1*, *Occludin*, and *Claudin-1* and the protein expression of Occludin and Claudin-1. This phenomenon may be due to the increased level of microbial metabolites such as lactate and SCFAs. Lactate plays a role in the development of mucosal barrier functions such as accelerating gut stem-cell-mediated epithelial development [42]. Further, SCFAs such as butyric acid maintain the tight junction of IECs [43]. Moreover, sIgA exerts the role of neutralizing the pathogens in the gut mucosa and maintaining the homeostasis of the gut microbiome [44]. We found that the level of sIgA in the colon of the SB group increased markedly after weaning, which may be due to the increased isobutyric acid and isovaleric acid altering IgA production by B cells in mice [45].

Furthermore, the bacteria in the gut lumen easily translocate to other organs due to the injury of the gut mucosal barrier [46]. Therefore, we detected the bacteria load in different organs including the liver, spleen, and kidney, and the results showed that *S. boulardii* early intervention significantly alleviated the bacteria load in the liver and had a trend to decrease the bacteria load in the spleen, which is similar to the finding that the bacterial translocation to mesenteric lymph nodes was reduced after supplementation with *S. boulardii* in early life piglets [47]. Meanwhile, the decrease in Potentially_Pathogenic such as *Shigella* and *unclassified_f__Enterobacteriaceae* may also contribute to alleviating the bacteria load in organs.

An emerging study demonstrated that early weaning contributes to a severe inflammatory response [48]. IL-1β, IL-6, and TNF-α are members of the pro-inflammatory cytokine family, which are mainly produced by innate immune cells because of the activation of the TLR/NF-κB pathway [49]. We found that *S. boulardii* early intervention significantly alleviated the content of IL-1β, IL-6, and TNF-α in the colon or serum. Moreover, there was a decrease in the relative mRNA expression of *TLR4* and *NF-κB* in the SB group. TLR4 senses LPS from Gram-negative bacteria and triggers the production of various pro-inflammatory mediators [50]. Thus, these results indicated that *S. boulardii* early intervention suppressed the inflammatory response possibly through the TLR4/NF-κB pathway by decreasing the relative abundance of Gram-negative bacteria after early weaning. Further, we also found that the relative mRNA expression of TGF-β and IL-10 were increased after weaning in the SB group, which can antagonize the pro-inflammatory effect of other cytokines, thereby maintaining gut mucosa homeostasis [51].

## 5. Conclusions

Collectively, early intervention by *S. boulardii* after birth not only modulated the gut microbiome by facilitating the beneficial bacteria colonization, resisting the pathogen bacteria colonization, maintaining the stability and complexity, and increasing SCFA and lactate levels but also enhanced the gut mucosal barrier function including elevating the number of goblet cells and mucin-related gene expression, promoting the protein and mRNA expression of tight junction and the secretion of sIgA, and suppressing the inflammatory response through the TLR4/NF-κB pathway.

## Figures and Tables

**Table 1:** Primers used for RT-PCR.

Gene	Primers Sequence (5′-3′)	Size (bp)	Accession No.
β-actin	F: GCAGGAGTACGATGAGTCCG R: ACGCAGCTCAGTAACAGTCC	74	NM_031144.3
MUC2	F: GCTGACGAGTGGTTGGTGAATG R: GATGAGGTGGCAGACAGGAGAC	135	XM_039101270.1
MUC3	F: ACTGCTTGTCCACGGATACTCA R: GACGGAGAACACAGCGAGGAT	140	XM_039090116.1
TFF3	F: GATAACCCTGCTGCTGGTCCTG R: CCACGGTTGTTACACTGCTCTG	150	NM_013042.2
ZO-1	F: GGCGTTCTAGAAGATAGCC R: GAAATCTACATTGTTCACCCTG	81	NM_001106266.1
Occludin	F: ACTATGAAACCGACTACACGA R: TGATAGGTGGATATTCCCTGAG	80	NM_031329.3
Claudin-1	F: GCTGTCATCGGGGGCATAAT R: CCTGGCCAAATTCATACCTGG	136	NM_031699.3
TGF-β	F: TGAGTGGCTGTCTTTTGACG R: CAGGAAGGGTCGGTTCATGT	196	NM_021578.2
IL-10	F: GCTCTTACTGGCTGGAGTGAG R: CTCAGCTCTCGGAGCATGTG	105	NM_012854.2
TLR4	F: TCCACAAGAGCCGGAAAGTT R: TGAAGATGATGCCAGAGCGG	126	NM_019178.2
NFκB	F: TTCAACATGGCAGACGACGA R: AGGTATGGGCCATCTGTTGAC	131	NM_001276711.1

MUC2, mucin2. MUC3, mucin2. TFF3, trefoil factor family 3. ZO-1, zonula occluden 1. TGF-β, transforming growth factor β. IL-10, interleukin 10. TLR4, toll-like receptor 4. NF-κB, nuclear factor kappa-light-chain-enhancer of activated B cells.

**Table 2:** Effects of *S. boulardii* early intervention on the growth performance of early-weaned rats.

Items	PBS	SB	*p*-Value
**1 day BW, g**	36.66 ± 3.13	36.91 ± 2.77	0.84
**3 day BW, g**	42.35 ± 2.1	42.7 ± 2.82	0.73
**7 day BW, g**	57.95 ± 2.56	60.18 ± 3.05	0.07
**1~3 d**			
**ADG, g/day**	1.90 ± 0.91	1.93 ± 0.85	0.93
**ADFI, g/day**	2.82 ± 0.80	2.93 ± 0.94	0.82
**FCR**	0.62 ± 0.15	0.67 ± 0.19	0.64
**3~7 day**			
**ADG, g/day**	3.9 ± 0.37	4.37 ± 0.52	0.02
**ADFI, g/day**	9.26 ± 1.03	9.03 ± 0.71	0.66
**FCR**	0.43 ± 0.06	0.49 ± 0.05	0.09
**1~7 day**			
**ADG, g/day**	3.04 ± 0.49	3.32 ± 0.52	0.19
**ADFI, g/day**	6.5 ± 0.55	6.42 ± 0.55	0.84
**FCR**	0.47 ± 0.05	0.52 ± 0.06	0.15

Values are presented as mean ± SEM, *n* = 12. 1 d, 3 d, and 7 d, day 1, 3, and 7 after weaning, respectively. PBS, rats early intervened with phosphate buffer solution. SB, rats early intervened with *S. boulardii*. BW, body weight. ADG, average daily gain. ADFI, average daily feed intake. FCR, feed conversion ratio.

**Table 3:** The network property parameters.

Parameters	PBS_W3	SB_W3	PBS_7day	SB_7day
Number of nodes	57	65	47	54
Number of edges	142	294	102	128
Average number of neighbors	4.912	8.730	4.489	4.741
Network density	0.088	0.138	0.102	0.089
Characteristic path length	4.360	2.963	3.545	3.997
Clustering coefficient	0.439	0.534	0.466	0.582
Network centralization	0.168	0.202	0.202	0.083

## Abbreviations

*S. boulardii*, *Saccharomyces boulardii*; PD, Postnatal day; CFUs, Colony-forming units; BW, Body weight; ADG, Average daily gain; ADFI, Average daily feed intake; FCR, Feed conversion ratio; OTUs, operational taxonomic units; PAS, Periodic acid Schiff; HE, Hematoxylin and eosin; qPCR, Quantitative real-time; Ct, Cycle thresholds; IL-1β, Interleukin 1β; IL-6, Interleukin 6; TNF-α, Tumor necrosis factor-α; sIgA, secretory immunoglobulin A; SEM, Standard error of mean; TLR4, toll-like receptor 4; NFκB, nuclear factor kappa-light-chain-enhancer of activated B cells; TGF-β, transforming growth factor β; IL-10, interleukin 10; PCoA, Principal coordinates analysis.
